# Copy number variation and neuropsychiatric problems in females and males in the general population

**DOI:** 10.1002/ajmg.b.32685

**Published:** 2018-10-11

**Authors:** Joanna Martin, Kristiina Tammimies, Robert Karlsson, Yi Lu, Henrik Larsson, Paul Lichtenstein, Patrik K. E. Magnusson

**Affiliations:** ^1^ Department of Medical Epidemiology & Biostatistics Karolinska Institutet Stockholm Sweden; ^2^ MRC Centre for Neuropsychiatric Genetics and Genomics Cardiff University Cardiff United Kingdom; ^3^ Center of Neurodevelopmental Disorders at Karolinska Institutet (KIND), Department of Women's and Children's Health Karolinska Institutet and Center for Psychiatry Research Stockholm Sweden; ^4^ School of Medical Sciences Örebro University Örebro Sweden

**Keywords:** anxiety, copy number variation, depression, neurodevelopmental problems, sex

## Abstract

Neurodevelopmental problems (NPs) are more common in males, whereas anxiety and depression are more common in females. Rare copy number variants (CNVs) have been implicated in neurodevelopmental disorders. The aim of this study was to characterize the relationship between rare CNVs with NPs, anxiety, and depression in a childhood population sample, as well as to examine sex‐specific effects. We analyzed a sample of *N* = 12,982 children, of whom 5.3% had narrowly defined NPs (clinically diagnosed), 20.9% had broadly defined NPs (based on validated screening measures, but no diagnosis), and 3.0% had clinically diagnosed anxiety or depression. Rare (<1% frequency) CNVs were categorized by size (100–500 kb or > 500 kb), type, and putative relevance to NPs. We tested for association of CNV categories with outcomes and examined sex‐specific effects. Medium deletions (OR[CI] = 1.18[1.05–1.33], *p* = .0053) and large duplications (OR[CI] = 1.45[1.19–1.75], *p* = .00017) were associated with broadly defined NPs. Large deletions (OR[CI] = 1.85[1.14–3.01], *p* = .013) were associated with narrowly defined NPs. There were no significant sex differences in CNV burden in individuals with NPs. Although CNVs were not associated with anxiety/depression in the whole sample, in individuals diagnosed with these disorders, females were more likely to have large CNVs (OR[CI] = 3.75[1.45–9.68], *p* = .0064). Rare CNVs are associated with both narrowly and broadly defined NPs in a general population sample of children. Our results also suggest that large, rare CNVs may show sex‐specific phenotypic effects.

## 1 INTRODUCTION

Neurodevelopmental problems (NPs) are psychiatric phenotypes that are characterized by childhood onset, high levels of comorbidity with other NPs, and other psychiatric conditions, and being more commonly diagnosed in males than females (Thapar, Cooper, & Rutter, 2017). Common diagnoses of NPs include attention‐deficit/hyperactivity disorder (ADHD), autism spectrum disorder (ASD), developmental coordination disorder (DCD), tic disorders, and intellectual disability (ID) (also referred to as learning difficulties). NPs are highly heritable, with a complex genetic architecture according to emerging genetic studies (de la Torre‐Ubieta, Won, Stein, & Geschwind, 2016; Faraone et al., 2015; O'Rourke, Scharf, Yu, & Pauls, 2009). Rare genetic variants, such as copy number variants (CNVs) and protein‐truncating variants have been implicated in clinically diagnosed ASD, ID, ADHD, DCD, and Tourette's syndrome (De Rubeis et al., 2014; Ganna et al., 2018; Girirajan et al., 2011; Huang et al., 2017; Mosca et al., 2016; Sanders et al., 2015; Williams et al., 2012). The contribution of rare variants to risk of anxiety and depression diagnoses in children has not been well characterized, with one recent population study finding no evidence of association (Guyatt et al., 2018).

Although genetic studies of NPs have tended to focus on categorically defined clinical disorders, evidence is emerging to suggest that these disorders reflect a quantitative extreme of continuously distributed traits in the general population. Twin studies suggest that ADHD, ASD, and mild ID share genetic risks with related traits in the population (Martin, Taylor, & Lichtenstein, 2017). Genome‐wide studies further demonstrate that common variants are shared to a degree between disorders and traits related to ADHD, ASD, and Tourette's syndrome (Darrow et al., 2017; Demontis et al., 2017; Robinson et al., 2016).

Evidence for shared rare variants across psychiatric disorders and related population traits or subthreshold psychiatric problems is also emerging. In a study of autism simplex families, adaptive and social‐communication difficulties in ASD probands and their unaffected siblings were associated with very rare de novo protein‐truncating variants, with no qualitative distinction between probands and siblings in this association (Robinson et al., 2016). A few studies have examined the manifestation of CNVs in adults in the general population, focusing on “neuropsychiatric” CNVs (i.e., loci robustly implicated in psychiatric disorders). These neuropsychiatric CNVs were associated with depression, lower scores on the General Assessment of Function scale, a history of reading and mathematical learning difficulties, lower cognitive abilities, and educational attainment in adults in the general population (Kendall et al., 2016; Männik et al., 2015; Stefansson et al., 2014). It appears that the same rare CNV loci are not only associated with multiple clinically diagnosed neuropsychiatric disorders (e.g., ASD, ID, schizophrenia, and ADHD [Girirajan et al., 2012; Marshall et al., 2016; Williams et al., 2012]) but also with neuropsychiatric and cognitive outcomes in adults in the general population (Kendall et al., 2016; Männik et al., 2015; Stefansson et al., 2014). The impact of CNVs on more broadly defined NPs in nonclinical childhood population samples has not been thoroughly examined. One recent population study of British children found associations between CNVs and cognitive measures (e.g., IQ) and ASD diagnoses but not anxiety or depression diagnoses or quantitative psychiatric traits of ADHD, ASD, or psychotic experiences (Guyatt et al., 2018). Further studies are needed to more fully characterize the impact of CNVs on childhood psychiatric problems.

One prominent characteristic of NPs is that they are more commonly diagnosed in males than females (Thapar et al., 2017). Population studies of childhood NPs also suggest that males score higher on measures of NPs than females (Larsson, Anckarsater, Råstam, Chang, & Lichtenstein, 2011; Lingam et al., 2010; Robinson, Lichtenstein, Anckarsäter, Happé, & Ronald, 2013). Although the reasons for this are still unclear, a few possible explanations have been proposed. First, it has been suggested that females are protected (e.g., by hormonal differences) from manifesting NPs and require a higher burden of risk to develop NPs. Evidence from several family studies seems to support this hypothesis (Beitchman, Hood, & Inglis, 1992; Martin, Walters, et al., 2018; Rhee & Waldman, 2004; Robinson et al., 2013; Smalley et al., 2000; Szatmari et al., 2012; Taylor et al., 2016), albeit other studies are inconsistent (Chen et al., 2017; Conti‐Ramsden, Falcaro, Simkin, & Pickles, 2007; Faraone, 2000; Goin‐Kochel, Abbacchi, & Constantino, 2007). Evidence from common variant analyses is also mixed, with the largest studies not finding differences in polygenic burden between males and females with ADHD and ASD (Hamshere et al., 2013; Martin, Hamshere, Stergiakouli, O'Donovan, & Thapar, 2014; Martin, Walters, et al., 2018; Mitra et al., 2016; Weiner et al., 2017). Conversely, females diagnosed with ASD or developmental delay appear to have an increased burden of rare disruptive CNVs and deleterious single‐nucleotide mutations compared with affected males (Gilman et al., 2011; Iossifov et al., 2012; Jacquemont et al., 2014; Levy et al., 2011; Neale et al., 2012; Pinto et al., 2014), though others have not found this effect for ultra‐rare protein‐truncating variants (Ganna et al., 2018). It is unknown whether rare CNVs are enriched in females compared to males in individuals affected with a broader range of NPs (e.g., ADHD and tic problems) in the general population.

Another possibility is that referral and diagnostic biases or sex‐specific manifestation of risk result in fewer females being diagnosed with NPs (Loomes, Hull, & Mandy, 2017; Quinn & Madhoo, 2014). It has been suggested that childhood psychiatric problems in females either manifest differently than in males or are interpreted differently by parents and teachers, for example, as anxiety or depression, instead of ADHD or ASD (Hull & Mandy, 2017; Quinn & Madhoo, 2014). If either of these are the case, one might expect that genetic risk factors relevant to NPs, such as rare CNVs, would be more strongly associated with such other psychiatric problems in females. This possibility has yet to be examined.

In this study, we first set out to test whether rare CNVs are associated with NPs (i.e., ADHD, ASD, motor problems, learning difficulties, and tic problems) and anxiety/depression in a population sample of children. Linkage with Swedish National Patient Registers and available study‐specific questionnaires allowed us to use both narrow (i.e., clinical diagnosis) and broad (i.e., screening measure) definitions of NPs, to distinguish between clinically recognized problems and subthreshold population phenotypic variation. Second, we tested whether there are any sex differences in CNV burden in the context of NPs or anxiety/depression in the population.

## 2 METHOD

### 2.1 Sample description

The Child and Adolescent Twin Study in Sweden (CATSS) is a population study of twin children born in Sweden since July 1992 (Anckarsäter et al., 2012). The study started in 2004 and since then has been the method of inviting twins into the Swedish Twin Registry (Magnusson et al., 2013). The CATSS cohort is being recruited when twins turn 9‐years old, but initially, 12‐year‐old twins were also recruited. Parents gave informed consent for study participation on behalf of their children. The study was approved by the Regional Ethical Review Board in Stockholm and Karolinska Institutet Ethical Review Board.

### 2.2 Phenotypic measures

Information on five specific NPs (ADHD, ASD, motor problems, learning difficulties, and tic problems) was obtained from two sources: life‐time registry‐based clinical diagnoses and screening via parental report at age 9 or 12 years old. Diagnoses for these NPs, as well as life‐time anxiety and depression, were obtained through linkage with the National Patient Register (NPR) in Sweden, using each individual's personal identification number. This NPR linkage contains information on all inpatient psychiatric care from 1987 to 2014 and outpatient consultations with specialists from 2001 to 2014. It includes best‐estimate specialist diagnoses according to International Classification of Diseases version 10 (ICD‐10) codes (WHO, 1993). Individuals were dichotomized based on presence or absence of each of the diagnoses. See Table 1 for a list of ICD codes and numbers of affected individuals.

**Table 1:** Phenotype descriptions

Phenotype	Narrowly defined psychiatric disorders		Broadly defined NPs^a^		Specific NPs^a^
	ICD diagnostic codes	*N* (%) affected	Description of A‐TAC measure	*N* (%) affected	Overall *N* (%) affected
ADHD	ADHD/hyperkinetic disorder (F90)	430 (3.3%)	19 items; screening cut‐off: ≥6	931 (7.2%)	1,361 (12.3%)
ASD	Autism (F84)	170 (1.3%)	17 items; screening cut‐off: ≥4.5	273 (2.1%)	443 (4.4%)
Motor problems	Motor disorders (F82)	22 (0.2%)	1 item; screening cut‐off: ≥0.5	864 (6.7%)	886 (8.4%)
Tic disorders	Tic disorders (F95)	42 (0.3%)	3 items; screening cut‐off: ≥1.5	384 (3.0%)	426 (4.2%)
Learning difficulties	ID (F70‐F79), language disorders (F80), scholastic disorders (F81)	233 (1.8%)	3 items; screening cut‐off: ≥1	1,641 (12.6%)	1,874 (16.2%)
Any NPs	Any of the above	689 (5.3%)	Any of the above	2,569 (20.9%)	3,258 (25.1%)
Anxiety	Social anxiety & phobias (F40), generalized anxiety and panic disorders (F41), separation anxiety and other childhood‐onset anxiety disorders (F93)	256 (2.0%)			
Depression	Single and recurrent major depressive disorders (F32‐F34)	220 (1.7%)			
Anxiety/depression	Either of the above	383 (3.0%)			

ADHD, attention‐deficit/hyperactivity disorder; ASD, autism spectrum disorder; A‐TAC, Autism‐Tics, ADHD, and Other Comorbidities inventory; ICD, International Classification of Diseases; ID: intellectual disability; NPs, neurodevelopmental problems.

*Note*. Diagnoses for each phenotype were collapsed into binary variables.

Excludes all individuals diagnosed with any narrowly defined NPs.

a Combined definition of either broadly or narrowly defined NPs for each of the specific phenotypes. Individuals may be affected with multiple specific NPs.

Study individuals were also screened for NPs using the Autism‐Tics, ADHD, and Other Comorbidities inventory (A‐TAC) (Hansson et al., 2005) administered to parents over the telephone when children were aged 9 or 12 years. This measure has been reported to have good to excellent sensitivity and specificity for predicting clinical diagnoses, using validated clinical cut‐offs (Larson et al., 2013). These validated cut‐offs were used to dichotomize children for each of the five NPs. Because ADHD, ASD, motor problems, learning difficulties, and tic problems show a high degree of comorbidity (Larson et al., 2013) (with substantial overlap also seen in CATSS, both for clinical ICD diagnoses and validated cut‐offs), we derived two summary measures based on meeting criteria for one or more of these NPs. Individuals receiving one or more ICD diagnosis of NPs were classed as having “narrowly defined NPs,” whereas individuals meeting any screening cut‐off based on the A‐TAC, but not receiving any of the clinical diagnoses, were classed as having “broadly defined NPs.” Presence of NPs using these two definitions were the primary outcomes in analyses. Individuals who did not meet criteria for either broadly or narrowly defined NPs were considered as the unaffected/control group, for all analyses. See Table 1 for details.

Separate variables were also derived for each of the five specific NPs, categorized using a combined definition of either broadly or narrowly defined NPs, as a result of limited power (see Table 1). It was possible for individuals to be categorized for more than one specific NP. A continuous measure of total NPs was derived by summing together the A‐TAC scales for all five NPs for each individual (range of scores: 0–43).

### 2.3 CNV calling and processing

DNA samples (from saliva) have been collected since 2008 from the participants after they are initially recruited to the study. DNA was extracted using the Chemagic STAR instrument from Hamilton Robotics and Puregene extraction kits. Samples were genotyped using the Illumina Infinium PsychArray‐24 BeadChip. For details of sample processing and standard genotype quality control (QC), see Brikell et al. (2018) and Supporting Information Text. For details of CNV calling and QC, see Supporting Information Text, and for an overview, see Supporting Information Figure S1. CNVs were called using the standard PennCNV (Wang et al., 2007) protocol. Strict CNV‐ and sample‐level QC was performed, following the protocol developed by the Psychiatric Genomics Consortium (Marshall et al., 2016). After all QC, the sample size for analysis consisted of *N* = 12,982 individuals, including *N* = 2,445 monozygotic and *N* = 3,554 dizygotic twin pairs.

### 2.4 CNV category definitions

CNV categories were defined based on size (all: >100 kb; medium: 100–500 kb; large: >500 kb), type (deletion or duplication), and presumed relevance to NPs (relevant CNV or other CNV). CNVs relevant to NPs were defined as those that either overlapped at least 50% with any of 69 genomic loci that have previously been implicated in ASD, ID, and/or schizophrenia (Coe et al., 2014; Marshall et al., 2016; Sanders et al., 2015) or with any gene within a set of 3,219 highly evolutionarily constrained genes that are intolerant to mutations (Lek et al., 2016). Further details about the definitions of these genomic regions can be found in the Supporting Information Text. CNVs that overlapped with these loci are referred to as neuropsychiatric disorder/evolutionarily constrained CNVs or “ND/EC CNVs” for short and CNVs that did not overlap are referred to as “other CNVs.” For all categories of CNVs, the number of CNV segments was categorized as “absent” if no CNV call had been made for an individual within the category or “present” if at least one called CNV met the criteria for that category.

### 2.5 Data analyses

For the primary analysis, we examined the overall association of all CNVs passing QC, with narrowly and broadly defined NPs, as well as with clinically defined anxiety/depression.

Several secondary tests were then performed to further examine the relationship between CNVs and these neuropsychiatric outcomes. First, CNVs were categorized by size and type to test the association of different CNV categories with each outcome. To determine whether the impact of rare CNVs differed in children with narrowly or broadly defined NPs, we compared the burden of each CNV category in relation to these outcomes. Next, we examined whether CNVs were associated with a continuous measure of NPs. We also examined whether significant associations for broad or narrow NPs were driven by specific NPs. Finally, we compared carriers of at least one ND/EC‐CNV and carriers of other CNVs to controls and to each other, to further characterize the origin of significant associations.

Several specific analyses were performed to investigate hypothesized sex‐specific CNV effects. First, we tested for an overall difference in CNV burden by sex in the whole sample. To test the hypothesis that females with NPs are carriers of a higher burden of risk variants as compared to affected males, we compared CNV burden by sex in individuals with NPs. To test the alternative hypothesis that genetic risk factors relevant to NPs are more likely to be associated with anxiety/depression in females than in males, we compared CNV burden by sex in individuals with any anxiety or depression diagnosis. We also tested the impact of comorbid clinically diagnosed NPs on this relationship by adjusting for the continuous measure of NPs. For all sex‐specific analyses, males were coded as “0” and females as “1.”

All analyses were performed using logistic or linear generalized estimating equations (with the package *drgee* [Zetterqvist & Sjölander, 2015] in R‐3.4.1), using family ID to cluster the data to account for related samples. The following covariates were included for all analyses: LRR SD, BAF SD, waviness factor, batch, and five principal components. Results were evaluated using an alpha level of 0.05.

## 3 RESULTS

Table 1 shows details of the psychiatric outcomes. There was a strong association between a child receiving an ICD diagnosis of any NP (narrowly defined NPs) and meeting study screening criteria for any NP (OR[CI] = 9.66[8.04–11.59], *p* = 1.2E‐130). *N* = 689 (5.3%) children had narrowly defined NPs, *N* = 2,569 children (19.8%) had broadly defined NPs (based on meeting screening cut‐offs but not receiving clinical diagnoses), and *N* = 9,724 (74.9%) children were unaffected by any definition of NPs. *N* = 383 (3.0%) children had clinically diagnosed anxiety and/or depression.

### 3.1 Association of CNVs with neuropsychiatric outcomes

There was an overall association between a child having any rare (<1% frequency) CNV >100 kb in size and having any broadly defined NPs (OR[CI] = 1.13[1.03–1.25], *p* = .014). There were no overall associations of presence of any CNVs with narrowly defined NPs (OR[CI] = 1.09[0.92–1.30], *p* = .31) or anxiety/depression diagnoses (OR[CI] = 1.15[0.92–1.42], *p* = .22).

#### 3.1.1 Secondary analyses

**Figure 1:**
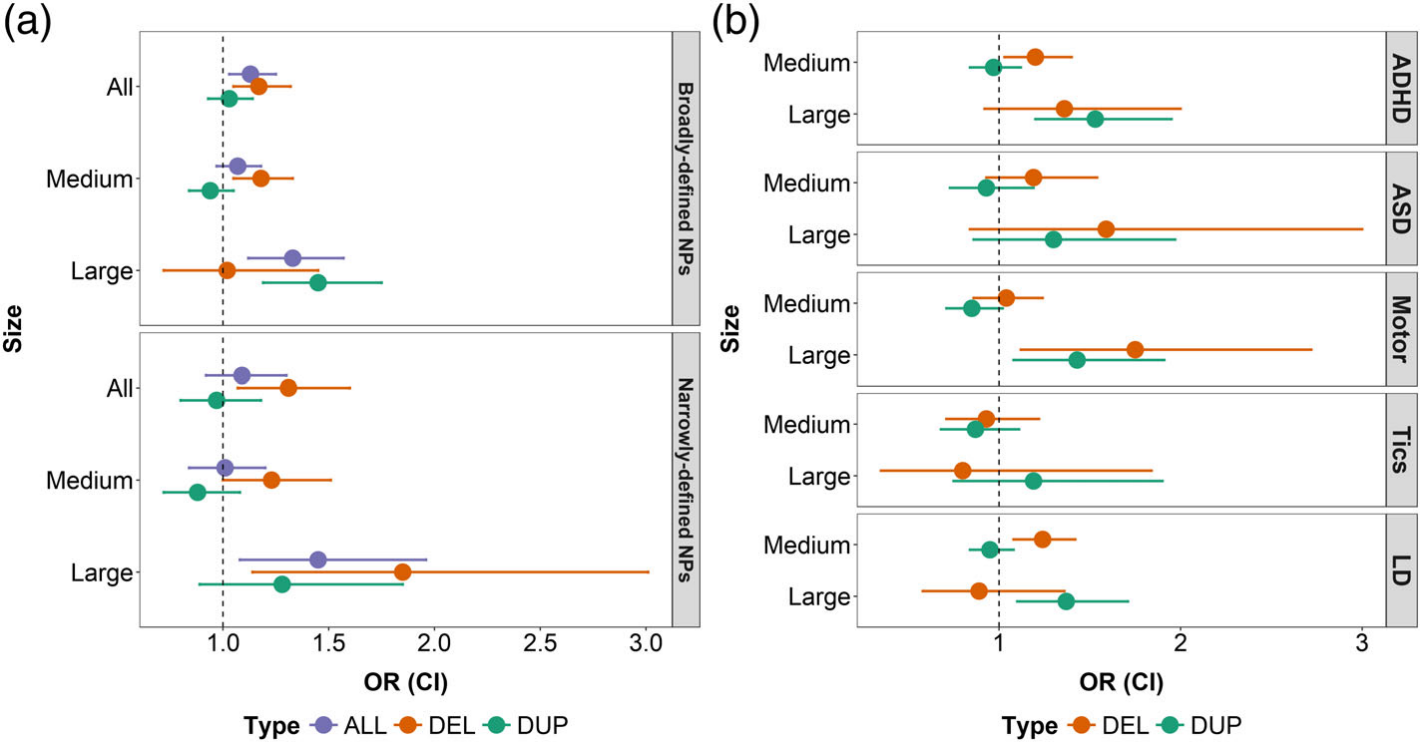
Association between presence of rare CNVs categorized by size (all: >100 kb; medium: 100–500 kb; large: >500 kb) and type (del: deletion; dup: duplication) with NPs, defined as: (a) broadly defined NPs (i.e., meeting criteria using study screening measures but not receiving clinical diagnoses) or narrowly defined NPs (i.e., receiving clinical diagnoses in the Swedish register data); and (b) specific NPs, using a combined definition of children meeting criteria for either broadly or narrowly defined NPs. Note that there is an overlap across the groups with some children affected with more than one NP. ADHD, attention deficit/hyperactivity disorder; ASD, autism spectrum disorder; CI, 95% confidence interval; LD, learning difficulties; OR, odds ratio [Color figure can be viewed at wileyonlinelibrary.com]

Associations between CNV categories of different sizes and types with NPs are shown in Figure 1a (see Supporting Information Table S1 for exact estimates). These secondary analyses indicated that medium‐sized (100–500 kb) deletions (OR[CI] = 1.18[1.05–1.33], *p* = .0053) and large (>500 kb) duplications (OR[CI] = 1.45[1.19–1.75], *p* = .00017) drove the observed overall association of CNVs with broadly defined NPs. Although there was no overall association between CNVs and narrowly defined NPs, large deletions did increase risk for this outcome (OR[CI] = 1.85[1.14–3.01], *p* = .013). There were no significant differences in presence of CNVs between children with narrowly or broadly defined NPs, except for large deletions (OR[CI] = 1.82[1.06–3.11], *p* = .028), which were more common in those with narrowly defined NPs (see Supporting Information Table S2). No CNV category was significantly associated with anxiety/depression; see Supporting Information Table S3.

CNVs were also associated with the continuous measure of total NPs (Table 2), with the strongest associations once again observed for medium deletions (beta[*SE*] = 0.30[0.11], *p* = .009) and large duplications (beta[*SE*] = 0.42[0.21], *p* = .046), suggesting that a medium‐sized CNV was associated with an average increase of 0.30 NP symptoms, and a large duplication was associated with an average increase of 0.42 NP symptoms. Excluding individuals with clinically diagnosed NPs reduced these effects (medium deletions: beta[*SE*] = 0.26[0.10], *p* = .0055; large duplications: beta[*SE*] = 0.27[0.17], *p* = .099); see Table 2.

**Table 2:** Association of presence of CNVs (categorized by size and type) with a continuous measure of NPs

CNV size	CNV type	Full sample (*N* = 12,982)		Excluding those with narrow NPs (*N* = 12,293)	
		Beta (*SE*)	*p*	Beta (*SE*)	*p*
All (>100 kb)	All	0.20 (0.09)	.0228	0.17 (0.07)	.0186
	Del	0.31 (0.11)	.00559	0.28 (0.09)	.00263
	Dup	0.08 (0.10)	.434	0.06 (0.08)	.487
Medium (100–500 kb)	All	0.16 (0.09)	.0817	0.15 (0.08)	.04
	Del	0.30 (0.11)	.00904	0.26 (0.10)	.0055
	Dup	−0.01 (0.10)	.922	0.02 (0.08)	.804
Large (>500 kb)	All	0.35 (0.18)	.0474	0.26 (0.15)	.0777
	Del	0.14 (0.30)	.643	0.24 (0.30)	.423
	Dup	0.42 (0.21)	.0461	0.27 (0.17)	.0994

CNVs, copy number variants; Del, deletions; Dup, duplications; NPs: neurodevelopmental problems.

Next, we examined the association of CNVs with specific NPs (see Figure 1b and Supporting Information Table S4). Medium deletions were significantly associated with ADHD (OR[CI] = 1.20[1.03–1.40], *p* = .017) and learning difficulties (OR[CI] = 1.24[1.08–1.42], *p* = .0017). Large duplications were associated with ADHD (OR[CI] = 1.53[1.20–1.95], *p* = .00051), motor problems (OR[CI] = 1.43[1.08–1.91], *p* = .013), and learning difficulties (OR[CI] = 1.37[1.10–1.71], *p* = .0047). Large deletions were also associated with motor problems (OR[CI] = 1.75[1.12–2.72], p = .013). Although the effect sizes for the association of CNV classes with ASD and large duplications with tics were similar to the analyses of ADHD and learning difficulties, these results did not reach significance (see Supporting Information Table S4).

**Figure 2:**
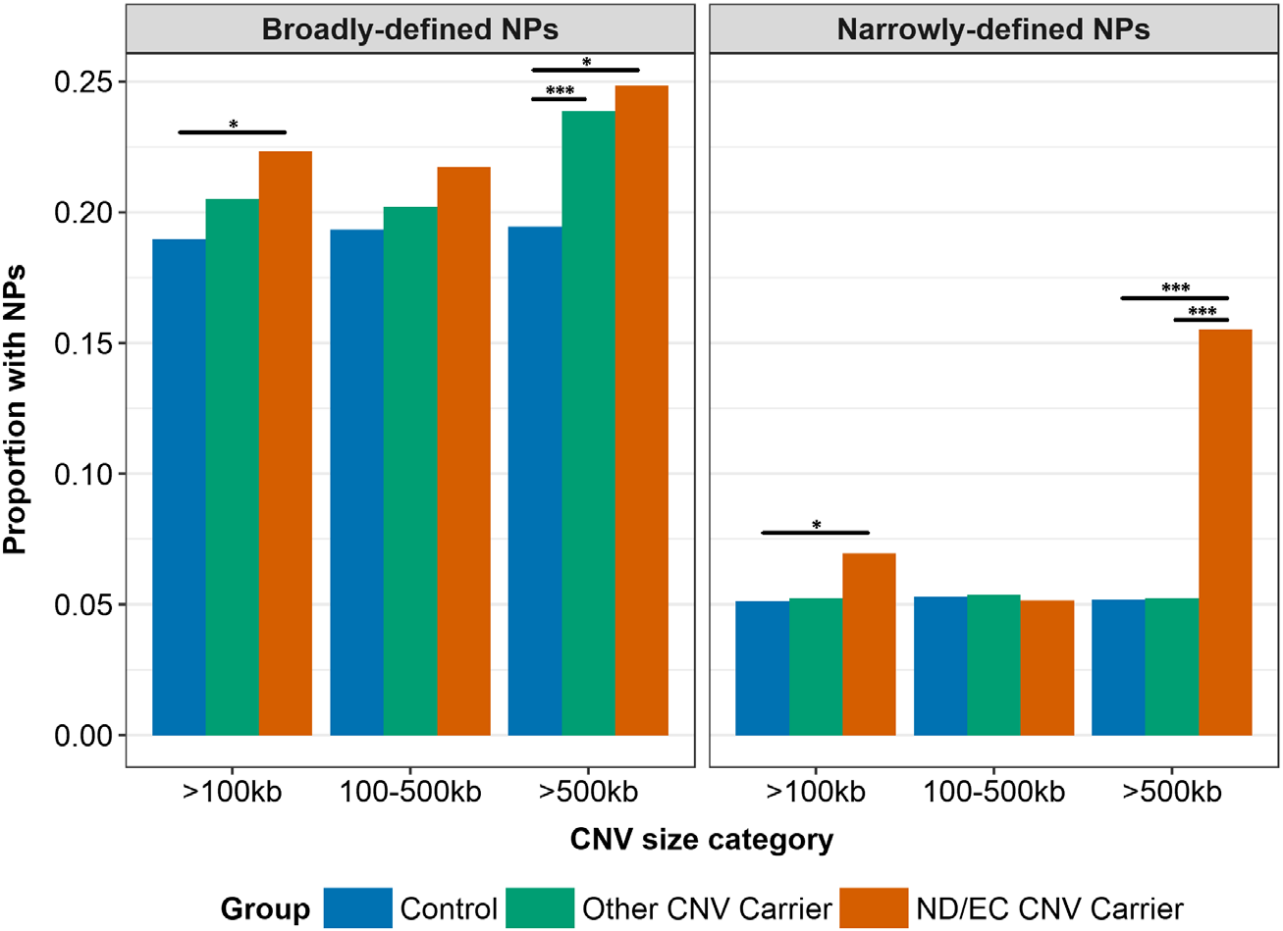
The proportion of broadly and narrowly defined NPs compared across carriers of CNVs that affect neuropsychiatric disorders or evolutionarily constrained genes (ND/EC CNVs), carriers of other CNVs, and controls who do not carry any CNVs, with CNVs stratified by size. *p < .05; **p < .01; ***p < .001 [Color figure can be viewed at wileyonlinelibrary.com]

When analyses were limited to individuals who are carriers of ND/EC CNVs (i.e., CNVs likely to be relevant to NPs) as compared with individuals without any CNVs, the observed effect sizes for large CNVs increased for both broadly (OR[CI] = 1.60[1.06–2.42], *p* = .025) and narrowly defined (OR[CI] = 3.64[2.16–6.13], *p* = 1.15E‐6) NPs (Supporting Information Table S5). Figure 2 shows the proportion of NPs in the different CNV carrier groups. Carriers of other (non‐ND/EC) large CNVs also had a higher proportion of broadly defined NPs as compared with noncarriers (OR[CI] = 1.28[1.06–1.54], *p* = .010). This association was nonsignificant for medium‐sized CNVs and for narrowly defined NPs as an outcome (Supporting Information Table S5). Conversely, carriers of large ND/EC CNVs had an increased risk of narrowly defined NPs compared to carriers of other large CNVs (OR[CI] = 3.65[1.83–7.27], *p* = 2.3E‐4); this effect was nonsignificant when examining medium‐sized CNVs or broadly defined NPs (Supporting Information Table S5).

### 3.2 Sex‐specific analyses

*N* = 1,486 (23.0%) males and *N* = 1,083 (16.6%) females met criteria for broadly defined NPs, *N* = 482 (7.5%) males and *N* = 207 (3.2%) females met criteria for narrowly defined NPs, and *N* = 157 (2.4%) males and *N* = 226 (3.5%) females met criteria for anxiety/depression; these sex differences were statistically significant (broad NPs: OR = 0.63[0.57–0.69], *p* = 7.4E‐22; narrow NPs: OR = 0.37[0.31–0.44], *p* = 6.6E‐27; anxiety/depression: OR = 1.44[1.16–1.79], *p* = 9.2E‐4).

There was no overall difference in presence of CNVs by sex; see Table 3. There were also no significant sex differences in CNV presence in children with narrowly or broadly defined NPs. Conversely, in those who had anxiety or depression diagnoses, females were significantly more likely than males to have large CNVs. The estimated effect size for this association increased when comorbid NPs were adjusted for, by including the continuous measure of NPs as a covariate in this analysis (Table 3).

**Table 3:** Associations of CNVs with sex

Sample	CNV size	CNV presence (%) in females/males	OR(CI)	*p*
Whole sample (*N* = 12,982)	All	43.9/44.4	0.99(0.91–1.08)	.85
	Medium	39.7/40.3	0.99(0.91–1.07)	.77
	Large	7.5/7.4	1.03(0.88–1.21)	.68
Broadly defined NPs (*N* = 2,569)	All	46.2/46.6	0.97(0.81–1.16)	.75
	Medium	40.7/41.8	0.96(0.80–1.15)	.65
	Large	9.4/8.7	1.06(0.78–1.44)	.73
Narrowly defined NPs (*N* = 689)	All	50.7/44.0	1.36(0.95–1.94)	.092
	Medium	43.5/38.8	1.24(0.86–1.78)	.26
	Large	10.1/9.5	1.18(0.62–2.22)	.61
Anxiety/depression (*N* = 383)	All	49.6/43.3	1.21(0.77–1.91)	.41
	Medium	41.2/40.1	0.96(0.61–1.52)	.87
	Large	12.4/3.8	3.75(1.45–9.68)	.0064
Anxiety/depression, adjusting for NPs (*N* = 383)	All	49.6/43.3	1.20(0.76–1.92)	.43
	Medium	41.2/40.1	0.93(0.58–1.48)	.76
	Large	12.4/3.8	4.28(1.71–10.73)	.0019

CI, 95% confidence interval; CNV, copy number variant; NPs, neurodevelopmental problems.

## 4 DISCUSSION

The results of this study add to the growing body of literature on the role of CNVs in psychiatric phenotypes. Our results are consistent with previous clinical studies of NPs, which reported significant enrichment of rare, deleterious CNVs in individuals with clinically diagnosed NPs (Girirajan et al., 2011; Huang et al., 2017; Mosca et al., 2016; Sanders et al., 2015; Williams et al., 2012). In this study, we extend this knowledge by also implicating rare CNVs in children with more broadly defined, subthreshold NPs and continuously assessed traits of broad NPs, which has not been shown before. We found no association between rare CNVs and clinically diagnosed anxiety and depression in this childhood sample, in line with a recent childhood population study (Guyatt et al., 2018), albeit in contrast to a population study which found an association between neuropsychiatric CNVs and depression (though not anxiety) in adults (Stefansson et al., 2014). Although we found no differences in CNV burden by sex in individuals with NPs, our results suggested that CNVs are enriched in females diagnosed with depression or anxiety, as compared to diagnosed males.

Several secondary analyses helped to characterize the role of CNVs in NPs and to pinpoint the categories of CNVs most relevant to NPs. Our results showed that narrowly defined NPs (i.e., real‐life clinical diagnoses independent of the study design) have specific associations with particularly large (>500 kb) CNVs, especially deletions and CNVs in regions previously implicated in other neuropsychiatric disorders and evolutionarily constrained, critical biological processes. Moreover, large deletions were enriched in narrowly defined NPs compared to broadly defined NPs. Conversely, medium‐sized (100–500 kb) deletions and large duplications were associated with broadly defined NPs. In addition to large CNVs that were likely to be relevant to NPs, large CNVs not affecting these regions were also relevant to broadly defined NPs. Thus, the evidence suggests that especially highly deleterious CNVs are associated with clinical diagnoses of NPs, whereas arguably somewhat less deleterious CNVs are associated with NPs defined more broadly in the population. This effect may be driven by larger CNVs having a more severe outcome as they disrupt more genes than medium‐sized CNVs, as well as a possible differential phenotypic impact of duplicated compared to deleted genetic material.

This study suggests that rare genetic variants related to clinically diagnosed disorders are also relevant to more broadly defined problems in the general population, consistent with previous twin and common variant studies (Martin et al., 2017). When examining NPs as a continuous distribution of symptoms, we saw a similar pattern of associations to when NPs were defined dichotomously, with attenuated, albeit largely consistent, effects when clinically diagnosed individuals were excluded (possibly as a result of reduced power). These results support the idea that neurodevelopmental disorders reflect the quantitative extreme of genetic factors operating dimensionally among individuals in the general population. These results are also consistent with studies adopting a genetics‐first approach, which have demonstrated that individuals with rare syndromes that are defined by specific rare CNVs (e.g., 22q11.2 deletion syndrome or Williams syndrome) show elevated levels of NPs, that do not always meet clinical diagnostic thresholds (Scerif & Baker, 2015; Tang, Antshel, Fremont, & Kates, 2015).

Multiple NPs were deliberately combined in this study because of the high comorbidity and shared genetic risk between different disorders (Lichtenstein, Carlström, Råstam, Gillberg, & Anckarsäter, 2010; Thapar et al., 2017). It has been suggested that a broad and heritable general psychopathology factor underlies NPs (Neumann et al., 2016; Pettersson, Larsson, & Lichtenstein, 2015). Our current results lend support to this idea by demonstrating that CNVs are to a large degree nonspecific, affecting multiple related neurodevelopmental outcomes in children from the general population. Although the analyses of specific NPs suggested that CNVs were most strongly associated with ADHD, motor problems and learning difficulties, these outcomes were in fact the most common ones in the sample (see Table 1); the estimated confidence intervals overlapped to a large degree for the different NPs and it is likely that these comparisons were affected by decreased power for ASD and tic problems. Thus, our results lend further evidence for pleiotropy of genetic risks impacting on multiple outcomes.

The second aim of our study was to determine whether CNVs showed sex‐specific associations that could elucidate the excessive male bias seen in NPs. In contrast to the previous clinical studies which reported an increased burden of large, rare CNVs in females with diagnosed ASD and developmental delay (Gilman et al., 2011; Jacquemont et al., 2014; Levy et al., 2011), our results showed no differences in CNV presence in males and females with NPs. This inconsistency may be because of ascertainment and measurement differences. The previous studies were clinically ascertained samples of children meeting research‐based diagnostic criteria for NPs, whereas the current dataset is a childhood population cohort study, with NPs defined using either a lifetime clinician's diagnosis (that is independent of study recruitment) or a broad study screening measure. Females diagnosed with ASD in clinical cohorts may be more severely affected and have more comorbid problems than affected males, which could also explain the discrepancy between previous studies and our results in this population sample (Gilman et al., 2011; Jacquemont et al., 2014; Levy et al., 2011).

Interestingly, we detected more large, rare CNVs in females with ICD diagnoses of anxiety or depression than in diagnosed males. This result could suggest that large, rare CNVs manifest differently in males and females. Adjusting for comorbid NPs (by including NP traits as a covariate) appeared to strengthen the association. This indicates that comorbid NPs in females with anxiety or depression do not explain the observed results. One interpretation is that large, rare CNVs may be more likely to be associated with anxiety or depression diagnoses in females. A complementary interpretation is that males with such CNVs may be less likely to be diagnosed with anxiety or depression; although the rate of large CNVs was similar by sex in the whole sample (females: 7.5%; males: 7.4%), in the individuals with diagnosed anxiety or depression, females had an increased rate (12.4%) whereas males had a decreased rate (3.8%) of large CNVs. In contrast to the male bias seen in NPs, epidemiological studies show a female bias toward diagnoses of anxiety and depression (Craske et al., 2017; Weissman et al., 1996). Given that the anxiety/depression diagnoses in this study were obtained from the study‐independent Swedish NPR and individuals were not comprehensively screened for these clinical diagnoses, our results hint that this prevalence difference may be in part as a result of sex‐specific referral or diagnostic biases, where female CNV carriers who manifest psychopathology and are referred for clinical assessment may be more likely to receive diagnoses of anxiety or depression rather than NPs, which are more common in males. A complementary finding was recently reported in the same dataset, whereby common risk alleles for ADHD are also enriched in females diagnosed with anxiety or depression as compared to affected males (Martin, Taylor, et al., 2018). However, the sample size of diagnosed individuals is limited in the current dataset and larger studies are needed to replicate and further interpret this novel finding.

The strengths of this study include the use of a large homogenous population sample with genome‐wide data as well as multiple assessments of NPs (based on study screening using parental questionnaires and study‐independent “real‐life” clinician's diagnoses). However, as this was a general population cohort, the proportion of individuals with clinical diagnoses was small, which of course constrains the power of the analyses. In particular, we were limited by sample size for analyzing specific NPs and thus broadly and narrowly defined problems were grouped to maximize power. As such, the classes of CNVs associated with specific NPs (e.g., medium deletions and large duplications associated with ADHD) may not generalize to associations only seen in clinically diagnosed disorders (Girirajan et al., 2011; Huang et al., 2017; Mosca et al., 2016; Sanders et al., 2015; Williams et al., 2012). The power was further limited for stratifying rare CNVs by size, type, and relevance to NPs, so we are unable to draw conclusions about the specific loci (and for the sex‐specific analyses even the category of CNVs) that may be driving observed associations. Another limitation is that certain types of CNVs and other structural variants (e.g., individuals with possible aneuploidies) were excluded from the study during QC, which may have affected the generalizability of our results to a broader class of rare structural variation in the general population.

In conclusion, this study demonstrates that rare CNVs not only impact on clinically recognized neurodevelopmental disorders in children but also on more broadly defined subthreshold and continuously defined NPs, which has not been reported previously in a nonclinical childhood population sample. Future studies using large genome‐wide datasets are needed to further determine the role of specific rare genomic loci in NPs and other psychiatric phenotypes in the general population. Our results add to studies of CNVs in adult populations (Kendall et al., 2016; Männik et al., 2015; Stefansson et al., 2014) and one recent childhood population study (Guyatt et al., 2018), demonstrating that population datasets with information on broad psychiatric problems will be an important addition to case–control studies in characterizing the role of rare variation in relation to neuropsychiatric phenotypes. Our study also finds evidence of a novel association between large, rare CNVs with anxiety and depression in females compared to males. This points to a possible role of sex‐specific manifestation of CNVs that would benefit from further study.

## CONFLICT OF INTEREST

Prof. Lichtenstein has served as a speaker for Medice, and Prof. Larsson has served as a speaker for Eli‐Lilly and Shire and has received research grants from Shire, all outside the submitted work. All other authors report no conflicts of interest.

## Supporting information

Appendix S1: Supplementary Materials
