# Supplementary material for: Copy number variation and neuropsychiatric problems in females and males in the general population
Source: Am J Med Genet B Neuropsychiatr Genet. 2018 Oct 11;180(6):341–50. doi: 10.1002/ajmg.b.32685 (PMC6767107; doi:10.1002/ajmg.b.32685)
Supplement: Supplementary file 1 — Appendix S1: Supplementary Materials [file AJMG-180-341-s001.docx]

# Supplementary Materials

## Supplementary Text

### Genotype quality control (QC)

DNA samples (from saliva) were genotyped on the Illumina Infinium PsychArray-24 BeadChip at the SNP&SEQ Technology Platform, Uppsala University, Uppsala, Sweden. Detailed QC information is available in (Brikell et al., 2018). All available dizygotic twins were genotyped and for most monozygotic twins, one in each pair of twins, was genotyped. For a subset of the twins, the zygosity was based on an algorithm using data on self- or parental-reported similarity.

The CATSS data were genotyped along with data from several other studies from the Swedish Twin Registry (Magnusson et al., 2013), including the studies TCHAD, RATSS, STOPPA and SALTY, in 18 batches. The QC filtered out 146 samples with less than 95% call rate or with unusual heterozygosity (autosomal inbreeding coefficient F outside +/-0.2). After merging genotypes of duplicate samples within and across batches (retaining only consensus calls; mismatch calls were set as missing), 18,193 samples with genotypes on 569,211 variants were available for further QC.

Single nucleotide polymorphism (SNP) QC excluded: 3,827 markers with call rate < 98%; 102 markers with over 10% discordant genotypes among 37 cross-batch duplicate samples; 323 markers with more than one discordant genotype among 84 pairs of MZ twins; 2,399 markers that failed Hardy-Weinberg Equilibrium test (p-value for testing HWE in all samples < 1e-6); 6 markers with large allele frequency differences from 1000 Genomes European samples (the absolute difference > 10%) and with mean GenCall scores<0.5; 35 common variants that were significantly associated with more than 1 genotyping batch (at p<5e-8).

Sample QC removed: 4 samples with call rate < 98%; 7 samples with unusual heterozygosity (autosomal inbreeding coefficient F outside +/- 0.2); 14 samples with possible sample contamination as indicated in the excessive relatedness with other samples (> 6 standard deviations from the mean of average sample relatedness in a random set of 1000 samples); 22 samples with sex violation (male with X-chr F < 0.5 or female with X-chr F >= 0.5); 248 samples identified as non-European ancestral outliers (> 6 standard deviations from the mean values of the first two principal components in 1000 Genomes European populations).

98% autosomal SNPs (545,733) and 98% samples (17,898) passed QC. In addition, ~99% of zygosity assumed a priori were confirmed by genomic relatedness. We also identified within-pair sample swap if both samples within any opposite-sex DZ pair or parent pair failed sex check. 6 out of 2255 (0.2%) opposite sex DZ pairs were corrected (i.e. swapped sample IDs and sex within pairs).

### Copy number variant (CNV) calling and processing

Genotypes were called using the GenCall algorithm in GenomeStudio 2011.1 (Illumina Inc.) with genotype cluster locations derived from the dataset. Log R Ratio (LRR) and B Allele Frequency (BAF) values were exported from GenomeStudio for further processing in PennCNV.

Raw intensity data were processed within genotyping batches. CNVs were called, using the PennCNV (Wang et al., 2007) (version 1.0.3) script ‘detect_cnv.pl’ following standard protocol and using default parameters. Only autosomal probes that passed previous SNP QC were included (see above). CNVs were called and initial QC steps were performed in the whole set of genotyped samples (N=18,560), which included samples from several different datasets not included in this study.

For an overview of CNV and sample-level QC, see Figure S1. The QC protocol followed that recently developed by the Psychiatric Genomics Consortium (Marshall et al., 2016), except that sample-specific cut-off thresholds were used. First, CNVs were joined if the distance separating two CNVs was less than 50% of their combined length, using a custom developed open source programme (Rees et al., 2016).

Further QC was restricted to samples passing SNP-based QC, which included removal of ancestry outliers; see above for details. Individuals were also excluded if they were outliers (>4 standard deviation (SD) away from the sample mean) on any of the following QC metrics generated by PennCNV: LRR SD>0.252, BAF SD>0.052, waviness factor>0.046, total number of segments (>383) and total CNV length (>42.1Mb). Note that additional filtering at stricter thresholds was also performed in the CATSS-only dataset (see later). We excluded individuals with possible aneuploidies (CNVs spanning >10% of any chromosome), which arise from a different mechanism (cell division) than other CNVs, and individuals with multiple instances of CNVs of opposite type called at the same loci.

Following exclusion of these poorly performing samples, CNV QC was performed. CNVs of opposite type (duplication and deletion) called at the same locus in an individual were excluded. CNVs spanning more than 50% of any of the following regions were excluded: centromeres, telomeres (100kb from ends of chromosomes), known segmental duplications, and immunoglobulin or T cell receptor loci. CNV loci occurring at >1% frequency (>176 CNVs) in the remaining set of samples were removed. CNV calls were also filtered out based on the following criteria: size (<100kb), coverage (<10 probes), probe density (>20kb/probe, calculated as size/probes per CNV) and PennCNV confidence score (<20).

The dataset was then restricted to individuals from the CATSS sample, excluding children with brain damage/injury, cerebral palsy, Down syndrome or reported chromosomal abnormalities. Where samples were genotyped multiple times, the run with the lower LRR SD was kept. Additional outliers were excluded based on the following QC metrics within the CATSS-only dataset: >4SD away from the sample mean for BAF SD or LRR SD, number of CNVs ≥10 and total CNV length >5Mb.

Samples originating from monozygotic (MZ) twin pairs that passed QC (N=71 twin pairs) were used to assess CNV concordance. 84/99 of CNVs>100kb in these samples were concordant (85% concordance rate) across twins and 15 were discordant (14 of which were <200kb). Visual inspection of BAF and LRR at these loci indicated poor support for true discordancy. Furthermore, recent analyses of a subset of the data detected no discordant calls across MZ twins (Stamouli et al., 2017). For each pair of MZ twins, the sample with the lower LRR SD was kept. This resulted in N=10,547 samples and N=6,533 rare CNVs>100kb. All CNV data were then imputed for pairs of MZ twins (N=2,445). An additional N=10 individuals were missing information from the A-TAC measure and were excluded. The final sample size for analysis was N=12,982 individuals, including N=2,445 MZ and N=3,554 DZ complete twin pairs.

### Definition of CNVs implicated in other neurodevelopmental disorders

CNV regions previously implicated in schizophrenia, autism spectrum disorder (ASD) and intellectual disability (ID) were obtained from published empirical studies. Regions implicated in schizophrenia came from a genome-wide study of CNVs in 21,094 schizophrenia cases and 20,227 controls; the list of 15 autosomal CNVs robustly associated with schizophrenia reported in Table 1 in this publication was used (Marshall et al., 2016). Regions implicated in ASD came from a family study of autism proband and parent trios; 12 de novo autosomal CNVs that were implicated in ASD using data from two independent studies (Simons Simplex Collection: N=2,591 trios and Autism Genome Project: N=2,096 trios), reported in Table 2 of the publication, were used (Sanders et al., 2015). Regions implicated in ID came from a study of 29,085 children with developmental delay and 19,584 healthy controls; 63 rare (population frequency <0.5%) autosomal CNVs that were significantly associated with developmental delay were used (Coe et al., 2014); genomic loci were extracted from eTable2 from a study utilising these data (Rees et al., 2016). For several disorders, CNVs of both types (duplication and deletion) were listed for a given locus and a number of regions overlapped across disorders. The merged list consisted of 69 unique genomic regions. All regions were converted to genome build hg19.

### Definition of highly evolutionarily-constrained gene regions

The set of highly evolutionarily constrained genes was defined using a metric for probability of a gene being loss-of-function (LoF) intolerant (pLI) based on the observed and expected protein-truncating variant (PTV) counts within each gene in a very large study of over 60,000 exomes (the Exome Aggregation Consortium; ExAC)(Lek et al., 2016). Publically available results based on the full ExAC dataset were downloaded from:

<ftp://ftp.broadinstitute.org/pub/ExAC_release/release0.3.1/functional_gene_constraint>

Genes with observed <10% of expected PTVs were deemed haploinsufficient or highly constrained. Genes with pLI ≥0.9 were selected as the set of highly constrained genes (3,219 genes). All gene regions were converted to build hg19.

### Principal components analysis

To adjust for population stratification, a principal components analysis was performed in PLINK after LD-pruning and removing genotyped autosomal SNPs located in long-range LD regions. Principal components (PCs) were calculated on unrelated individuals and projected onto relatives. The first 5 principal components were included as covariates in all analyses.

### References

Brikell, I., Larsson, H., Lu, Y., Pettersson, E., Chen, Q., Kuja-Halkola, R., Karlsson, R., et al. (2018). The contribution of common genetic risk variants for ADHD to a general factor of childhood psychopathology. *Molecular Psychiatry*.

Coe, B. P., Witherspoon, K., Rosenfeld, J. A., van Bon, B. W. M., Vulto-van Silfhout, A. T., Bosco, P., Friend, K. L., et al. (2014). Refining analyses of copy number variation identifies specific genes associated with developmental delay. *Nature Genetics*, *46*(10), 1063–1071.

Lek, M., Karczewski, K. J., Minikel, E. V., Samocha, K. E., Banks, E., Fennell, T., O’Donnell-Luria, A. H., et al. (2016). Analysis of protein-coding genetic variation in 60,706 humans. *Nature*, *536*(7616), 285–291. Nature Research.

Magnusson, P. K. E., Almqvist, C., Rahman, I., Ganna, A., Viktorin, A., Walum, H., Halldner, L., et al. (2013). The Swedish Twin Registry: establishment of a biobank and other recent developments. *Twin research and human genetics : the official journal of the International Society for Twin Studies*, *16*(1), 317–29. Cambridge University Press.

Marshall, C. R., Howrigan, D. P., Merico, D., Thiruvahindrapuram, B., Wu, W., Greer, D. S., Antaki, D., et al. (2016). Contribution of copy number variants to schizophrenia from a genome-wide study of 41,321 subjects. *Nature Genetics*, *49*(1), 27–35. Nature Research.

Rees, E., Kendall, K., Pardiñas, A. F., Legge, S. E., Pocklington, A., Escott-Price, V., MacCabe, J. H., et al. (2016). Analysis of Intellectual Disability Copy Number Variants for Association With Schizophrenia. *JAMA Psychiatry*, *73*(9), 963. American Medical Association.

Sanders, S. J., He, X., Willsey, A. J., Ercan-Sencicek, A. G., Samocha, K. E., Cicek, A. E., Murtha, M. T., et al. (2015). Insights into Autism Spectrum Disorder Genomic Architecture and Biology from 71 Risk Loci. *Neuron*, *87*(6), 1215–1233.

Stamouli, S., Anderlid, B.-M., Willfors, C., Thiruvahindrapuram, B., Wei, J., Berggren, S., Nordgren, A., et al. (2017). Copy Number Variation Analysis of 100 Twin Pairs Enriched for Neurodevelopmental Disorders. *Twin Research and Human Genetics*, *In Press*.

Wang, K., Li, M., Hadley, D., Liu, R., Glessner, J., Grant, S. F., Hakonarson, H., et al. (2007). PennCNV: an integrated hidden Markov model designed for high-resolution copy number variation detection in whole-genome SNP genotyping data. *Genome Research*, *17*(11), 1665–1674.

## Table S1: Association of presence of CNVs (categorized by size and type) with narrowly- and broadly-defined neurodevelopmental problems

| CNV Size | CNV Type | Rate of CNVs in controls (%) | Broadly-defined NPs | | | Narrowly-defined NPs | | |
| --- | --- | --- | --- | --- | --- | --- | --- | --- |
|  |  |  | **Rate of CNVs (%)** | **OR (CI)** | **P** | **Rate of CNVs (%)** | **OR (CI)** | **p** |
| All  (>100kb) | **All** | 43.4 | 46.4 | 1.13 (1.03-1.25) | 0.0139 | 46.0 | 1.09 (0.92-1.30) | 0.312 |
|  | **Del** | 20.7 | 23.2 | 1.17 (1.05-1.32) | 0.0062 | 25.3 | 1.31 (1.07-1.60) | 0.00891 |
|  | **Dup** | 28.8 | 29.6 | 1.03 (0.93-1.14) | 0.594 | 28.7 | 0.97 (0.80-1.18) | 0.756 |
| Medium (100-500kb) | **All** | 39.6 | 41.3 | 1.07 (0.97-1.18) | 0.165 | 40.2 | 1.01 (0.84-1.20) | 0.934 |
|  | **Del** | 19.4 | 22.0 | 1.18 (1.05-1.33) | 0.00532 | 22.8 | 1.23 (1.00-1.51) | 0.0502 |
|  | **Dup** | 25.2 | 24.3 | 0.94 (0.84-1.05) | 0.282 | 23.4 | 0.88 (0.72-1.08) | 0.215 |
| Large (>500kb) | **All** | 6.8 | 9.0 | 1.33 (1.12-1.57) | 0.00138 | 9.7 | 1.45 (1.08-1.96) | 0.0147 |
|  | **Del** | 1.8 | 1.9 | 1.02 (0.72-1.45) | 0.898 | 3.3 | 1.85 (1.14-3.01) | 0.0125 |
|  | **Dup** | 5.1 | 7.3 | 1.45 (1.19-1.75) | 0.000173 | 6.5 | 1.28 (0.89-1.85) | 0.179 |

CI: 95% confidence interval; CNVs: copy number variants; Del: deletions; Dup: duplications.

## Table S2: Association of presence of CNVs (categorized by size and type) with definition of neurodevelopmental problems

| CNV Size | CNV Type | OR (CI) | p |
| --- | --- | --- | --- |
|  |  |  |  |
| All  (>100kb) | **All** | 0.98 (0.82-1.19) | 0.863 |
|  | **Del** | 1.12 (0.90-1.38) | 0.302 |
|  | **Dup** | 0.96 (0.78-1.18) | 0.71 |
| Medium (100-500kb) | **All** | 0.96 (0.79-1.15) | 0.637 |
|  | **Del** | 1.05 (0.84-1.30) | 0.689 |
|  | **Dup** | 0.95 (0.77-1.18) | 0.675 |
| Large (>500kb) | **All** | 1.09 (0.79-1.51) | 0.583 |
|  | **Del** | 1.82 (1.06-3.11) | 0.0288 |
|  | **Dup** | 0.88 (0.60-1.30) | 0.533 |

CNVs: copy number variants; Del: deletions; Dup: duplications. Children with broadly-defined neurodevelopmental problems (NPs) are coded as ‘0’ and those with narrowly-defined NPs are coded as ‘1’.

## Table S3: Results of association of CNV presence (categorized by size and type) with clinically-diagnosed anxiety/depression

| CNV Size | CNV Type | Rate of CNVs (%) | | OR (CI) | P |
| --- | --- | --- | --- | --- | --- |
|  |  | **Affected** | **Unaffected** |  |  |
| All | **All** | 47 | 44 | 1.15 (0.92-1.42) | 0.215 |
|  | **Del** | 23.2 | 21.4 | 1.14 (0.88-1.48) | 0.317 |
|  | **Dup** | 31.3 | 28.9 | 1.14 (0.90-1.44) | 0.288 |
| Medium (100-500kb) | **All** | 40.7 | 40 | 1.05 (0.84-1.31) | 0.683 |
|  | **Del** | 21.7 | 20 | 1.13 (0.86-1.48) | 0.381 |
|  | **Dup** | 25.8 | 24.9 | 1.06 (0.82-1.36) | 0.662 |
| Large (>500kb) | **All** | 8.9 | 7.4 | 1.28 (0.88-1.86) | 0.189 |
|  | **Del** | 1.8 | 1.9 | 0.98 (0.46-2.11) | 0.961 |
|  | **Dup** | 7.3 | 5.5 | 1.41 (0.93-2.12) | 0.102 |

CI: 95% confidence interval; CNVs: copy number variants; Del: deletions; Dup: duplications.

## Table S4: Association of presence of CNVs (categorized by size and type) with specific neurodevelopmental problems

| Phenotype | CNV Size | CNV Type | Rate of CNVs (%) | | OR (CI) | p |
| --- | --- | --- | --- | --- | --- | --- |
|  |  |  | **Controls** | **Affected** |  |  |
| ADHD | **Medium** | **Del** | 19.4 | 22.3 | 1.20(1.03-1.40) | 0.0168 |
|  |  | **Dup** | 25.2 | 25 | 0.97(0.84-1.12) | 0.65 |
|  | **Large** | **Del** | 1.8 | 2.6 | 1.36(0.92-2.00) | 0.128 |
|  |  | **Dup** | 5.1 | 7.6 | 1.53(1.20-1.95) | 0.000512 |
| ASD | **Medium** | **Del** | 19.4 | 22.3 | 1.19(0.93-1.54) | 0.166 |
|  |  | **Dup** | 25.2 | 24.4 | 0.93(0.73-1.19) | 0.563 |
|  | **Large** | **Del** | 1.8 | 2.9 | 1.59(0.84-3.00) | 0.155 |
|  |  | **Dup** | 5.1 | 6.5 | 1.30(0.86-1.97) | 0.216 |
| Motor problems | **Medium** | **Del** | 19.4 | 20 | 1.04(0.86-1.24) | 0.708 |
|  |  | **Dup** | 25.2 | 22.3 | 0.85(0.71-1.02) | 0.0839 |
|  | **Large** | **Del** | 1.8 | 3 | 1.75(1.12-2.72) | 0.0132 |
|  |  | **Dup** | 5.1 | 7.2 | 1.43(1.08-1.91) | 0.0132 |
| Tic problems | **Medium** | **Del** | 19.4 | 18.1 | 0.93(0.71-1.22) | 0.607 |
|  |  | **Dup** | 25.2 | 23.2 | 0.87(0.68-1.11) | 0.268 |
|  | **Large** | **Del** | 1.8 | 1.4 | 0.80(0.35-1.84) | 0.599 |
|  |  | **Dup** | 5.1 | 6.1 | 1.19(0.75-1.90) | 0.462 |
| LD | **Medium** | **Del** | 19.4 | 22.8 | 1.24(1.08-1.42) | 0.00171 |
|  |  | **Dup** | 25.2 | 24.6 | 0.95(0.84-1.08) | 0.455 |
|  | **Large** | **Del** | 1.8 | 1.6 | 0.89(0.58-1.36) | 0.59 |
|  |  | **Dup** | 5.1 | 7 | 1.37(1.10-1.71) | 0.00471 |

CI: 95% confidence interval; CNVs: copy number variants; Del: deletions; Dup: duplications; ADHD: attention deficit hyperactivity disorder, ASD: autism spectrum disorder; LD: learning difficulties. Medium: 100-500kb; large: >500kb. N.B. a sub-group of children are affected with more than one neurodevelopmental outcome.

Combined definition of either broadly- or narrowly-defined NPs for each of the specific phenotypes. Individuals may be affected with multiple specific NPs.

## Table S5: Association of NPs with CNVs categorized by putative relevance to NPs

| CNV | Comparison | Broadly-defined NPs | | Narrowly-defined NPs | | |
| --- | --- | --- | --- | --- | --- | --- |
|  |  | **OR(CI)** | **p** | **OR(CI)** | **p** |  |
| All  (>100kb) | **ND/EC vs. control** | 1.24(1.03-1.49) | 0.0209 | 1.44(1.05-1.99) | 0.0243 |  |
|  | **Other vs. control** | 1.11(1.00-1.23) | 0.0508 | 1.03(0.85-1.23) | 0.774 |  |
|  | **ND/EC vs. other** | 1.14(0.95-1.38) | 0.161 | 1.35(0.97-1.88) | 0.0722 |  |
| Medium (100-500kb) | **ND/EC vs. control** | 1.14(0.93-1.38) | 0.203 | 1.01(0.69-1.47) | 0.978 |  |
|  | **Other vs. control** | 1.06(0.96-1.18) | 0.273 | 1.01(0.84-1.21) | 0.94 |  |
|  | **ND/EC vs. other** | 1.09(0.89-1.33) | 0.403 | 0.94(0.64-1.40) | 0.77 |  |
| Large (>500kb) | **ND/EC vs. control** | 1.60(1.06-2.42) | 0.025 | 3.64(2.16-6.13) | 1.15E-6 |  |
|  | **Other vs. control** | 1.28(1.06-1.54) | 0.00998 | 1.07(0.75-1.53) | 0.702 |  |
|  | **ND/EC vs. other** | 1.31(0.81-2.13) | 0.267 | 3.65(1.83-7.27) | 2.32E-4 |  |

CNVs: copy number variants; NPs: neurodevelopmental problems; CI: 95% confidence interval; ND/EC: neuropsychiatric disorder/evolutionarily-constrained copy number variant.

## Figure S1: Detailed overview of sample- and CNV-level quality control


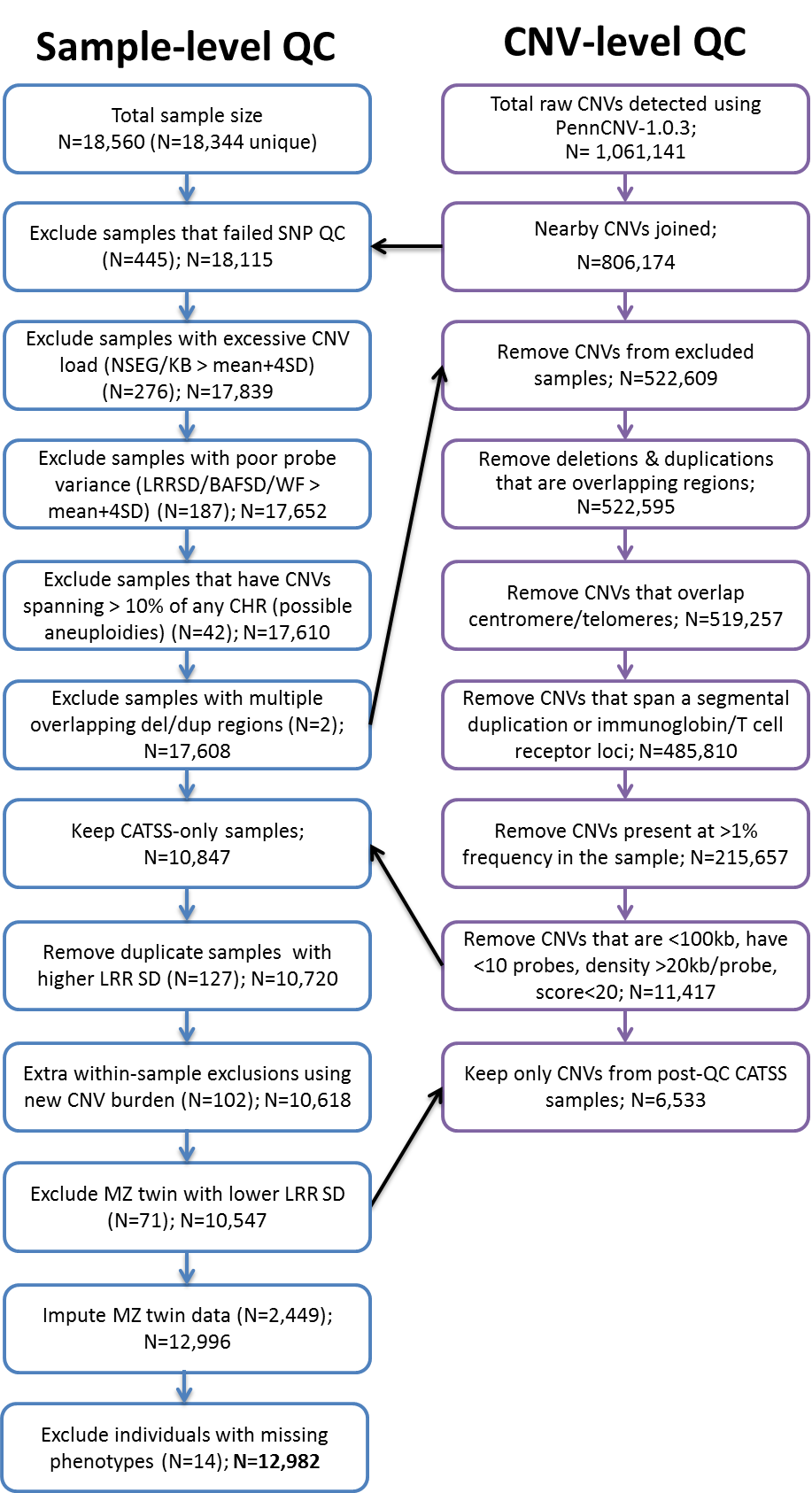


QC: quality control; CNV: copy number variant; SNP: single nucleotide polymorphism; LRRSD: Log R Ratio; BAFSD: B Allele Frequency; WF: waviness factor; CATSS: Child and Adolescent Twin Study in Sweden.
